# Clove Essential Oil as an Alternative Approach to Control Postharvest Blue Mold Caused by *Penicillium italicum* in Citrus Fruit

**DOI:** 10.3390/biom9050197

**Published:** 2019-05-21

**Authors:** Chuying Chen, Nan Cai, Jinyin Chen, Chunpeng Wan

**Affiliations:** 1 Jiangxi Key Laboratory for Postharvest Technology and Nondestructive Testing of Fruits & Vegetables, Jiangxi Agricultural University, Nanchang 330045, China; cy.chen@jxau.edu.cn (C.C.); wq1252733770@163.com (N.C.); 2 Pingxiang University, Pingxiang 337055, China

**Keywords:** blue mold, clove essential oil, induced disease resistance, *Penicillium italicum*

## Abstract

*Penicillium italicum* causes blue mold disease and leads to huge economic losses in citrus production. As a natural antifungal agent, clove essential oil (CEO), which is a generally recognized as safe (GRAS) substance, shows strong in vitro activity against fungal pathogens. However, few studies on CEO for controlling postharvest blue mold disease caused by *P. italicum* in citrus fruit have been reported. Our aims were to investigate the control efficacy and possible mechanisms involved of CEO against *P. italicum*. In the present study, CEO treatment inhibited the disease development of blue mold when applied at 0.05% to 0.8% (*v/v*), and with the effective concentration being obtained as 0.4% (*v/v*). Besides its direct antifungal activity, CEO treatment also spurred a rapid accumulation of H_2_O_2_ compared with untreated fruits, which might contribute to enhancing an increase in the activities of defense-related enzymes, such as β-1,3-glucanase (β-Glu), chitinase (CHI), phenylalanine ammonia-lyase (PAL), peroxidase (POD), polyphenol oxidase (PPO), and lipoxygenase (LOX) in citrus fruit. Results of real time-quantitative polymerase chain reaction (RT-qPCR) showed that the gene expressions of β-Glu, CHI, PAL, POD and PPO were up-regulated in CEO-treated fruits. At the same time, CEO treatment led to down-regulated expression of the LOX gene in citrus fruit. Clove essential oil effectively control the disease incidence of blue mold decay in citrus fruit by motivating the host-defense responses, suppressing the malondialdehyde (MDA) accumulation while enhancing the activities and gene expressions of defense-related enzymes. Our study provides an alternative preservative applying CEO to reduce postharvest fungal decay in citrus fruit.

## 1. Introduction

Citrus fruit is an important fruit and is highly popular all over the world due to its sweet, juicy, desirable flavor as well as its abundant nutrition [1]. However, due to its tender peel and rich nutrition, citrus fruit is also susceptible to fungal pathogens infection particularly after harvest, which can lead to massive economic losses and even to serious environmental pollution [2]. Among postharvest diseases, blue mold rot, caused by *Penicillium italicum*, is a serious postharvest disease and accounts for up to 20–50% of fruit decay in China [3]. Currently, the use of synthetic fungicides (e.g. imazalil, prochloraz, and thiabendazole) is the most effective and simplest approach for diseases management in the citrus postharvest industry. However, some synthetic fungicides are restricted due to the growing public concerns regarding chemical residues on human health and environmental contamination, as well as the appearance of fungicide-resistant pathogens [4]. Accordingly, natural alternative preservatives for the control of postharvest fungal diseases in citrus fruit during storage, transportation, and marketing are eagerly required. Therefore, in the past few decades, researchers worldwide have begun to pay their attention towards applying natural plant oils such as bergamot (*Citrus bergamia*), cinnamon (*Cinnamomum zeylanicum* L.), clove (*Syzygium aromaticum* L.), sage (*Salvia officinalis* L.), oregano (*Origanum vulgare* L.), tea tree (*Melaleuca alternifolia* L.), and thyme (*Tymus vulgaris* L.) to control pathogens infection and reduce postharvest fungal rot in fresh horticultural products [5,6,7,8,9,10,11,12]. 

Essential oils (EOs) are aromatic and volatile oily liquids extracted by hydrodistillation or supercritical fluid extraction from plants and spices, which are abundant in bioactive compounds with antimicrobial and antioxidant properties [8,13,14,15]. Several studies have been reported the in vitro antifungal properties of EOs against postharvest fungal pathogens, such as *Penicillium* spp. [5,6,7,9,16,17], *Alternaria* spp. [7,9,18], *Colletotrichum* spp. [9,11,19,20], *Aspergillus* spp. [7,10,21], and *Botrytis cinerea* [22,23,24]. Among EOs, clove essential oil (CEO) has been well known and used for antimicrobial, fungicidal, antiviral, antioxidant, antitumor, anesthetic, insecticidal, and cosmetic applications [25]. In the last two decades, growing interest has been focused on CEO in place of synthetic chemical fungicides to reduce postharvest diseases rot and improve the storage quality of horticultural products because of its potent antimicrobial, antifungal, and antioxidant activity [9,10,12,26]. 

In our previous study, we have found that CEO has strong antifungal activity against *P. italicum*, *Penicillium digitatum*, *Alternaria citri* and *Geotrichum citri-aurantii* in citrus fruit [9]. Although the in vitro antifungal activity of CEO against *P. italicum* was widely explored, only few published works focused on the control efficacy of CEO as a natural antifungal agent towards postharvest blue mold in citrus fruit and its mechanisms remained unknown. Therefore, the aim of this research was to evaluate the control efficacy of CEO in inhibition postharvest citrus blue mold decay caused by *P. italicum*, and the possible defense mechanisms involved, inducing the antifungal efficacy of CEO against *P. italicum* in citrus fruit wounds, the response of reactive oxygen species (ROS), the activities and gene expressions of defense-related enzymes.

## 2. Materials and Methods 

### 2.1. Clove Essential Oil and Pathogens

The commercially available CEO obtained from *S. aromaticum* L. was provided from Shanghai Huien international business Co., Ltd. (Shanghai, China), originated from Sri Lanka, and was stored in dark bottles at 4 °C prior to use.

The tested pathogen of *P. italicum* was isolated from an infected citrus fruit with the typical blue mold symptoms and grown on potato dextrose agar (PDA: peeled potatoes, 200 g; glucose, 20 g; agar, 18 g; distilled water, 1000 mL) medium at 25 °C. The spore suspension was prepared by scraping a 7-day-old pure culture of *P. italicum* using a sterile loop and adjusted to 5 × 10^4^ spore/mL using a hemocytometer (Hausser scientific, Horsham, PA, USA).

### 2.2. In Vivo Antifungal Assay

The citrus fruit (*Citrus reticulata* Blanco cv. Xinyu tangerines) was harvested at commercial maturity from a local orchard located in Xinyu City, China and those that were healthy, had a consistent size (72–84 g), a uniform color (citrus color index, 3.6–4.5), and were free of bruises or disease were chosen as the experimental material. The in vivo test was carried out according to our previous research [9]. All selected fruits were dipped in 1% (*v/v*) sodium hypochlorite solution for 2 min and were then washed with running tap water to remove the residual disinfectant and air-dried in a sterilized bechtop (SW-CJ-2DF, Suzhou purification equipment Co. Ltd., Suzhou, China) before wounding.

A uniform wound (4 mm diameter, 2 mm deep) was made with a sterile puncher at the equatorial side per fruit. Then, 20 μL of CEO emulsion at different concentrations (0, 0.05, 0.1, 0.2, 0.4 and 0.8%) was injected into each wound. After 30 min, 20 μL of *P. italicum* spore suspension (5 × 10^4^ spore/mL) was reinjected into each wound. Both CEO-treated and control fruits were placed in containers (35 cm × 26 cm polyethylene-lined plastic boxes) at 25 °C. The lesion diameter was measured with a vernier caliper at 3, 5, and 7 days post inoculation. The disease severity was observed by evaluating the disease development of blue mold using the following scale: scale 0, lesion diameter = 0 mm (no decay); scale 1, 1 mm ≤ lesion diameter ≤ 10 mm; scale 2, 10 mm < lesion diameter ≤ 20 mm; scale 3, 20 mm < lesion diameter ≤ 40 mm; scale 4, lesion diameter > 40 mm. The disease severity was calculated using the following formula:

$$\mathrm{Disease}\;\mathrm{severity}\;(\%)\;=\;\frac{\sum \mathrm{disease}\;\mathrm{scale}\times \mathrm{number}\;\mathrm{of}\;\mathrm{fruit}\;\mathrm{in}\;\mathrm{each}\;\mathrm{scale}}{\mathrm{highest}\;\mathrm{disease}\;\mathrm{scale}\;\times \;\mathrm{number}\;\mathrm{of}\;\mathrm{total}\;\mathrm{fruit}}\times 100$$

### 2.3. Clove Essential Oil Treatment and Sample Collection

The sterilized fruits were wounded (4 mm diameter, 2 mm deep) with two wounds symmetrically made at the equatorial side per fruit. Then, 20 μL of CEO emulsion (0.4%) or emulsion without CEO as the control was injected into each wound. After reinjecting 20 μL of *P. italicum* spore suspension (5 × 10^4^ spore/mL), the CEO treated and control fruits were maintained at 25 °C. Tissue samples of healthy peel at 5–15 mm from the edge of the inoculated lesion from ten fruits in each replicate were collected at 0, 12, 24, 48, 72 and 96 hours post inoculation (hpi), immediately frozen in liquid nitrogen for 3 min, and kept at −80 °C for the biochemical analysis.

### 2.4. Assay of Hydrogen Peroxide (H_2_O_2_) Content

Frozen peel samples (2.0 g fresh weight, stemmed from the CEO treated or control fruits) were homogenized in 5 mL of ice-cold acetone and then centrifuged at 12,000 × g for 30 min at 4 °C (Hettich Universal-320R, Hettich, Frankenberg, Germany). The supernatants were collected and used for the assay of H_2_O_2_ content. H_2_O_2_ content was determined by applying a H_2_O_2_ assay kit (Jiancheng Bioeng. Inst., Nanjing, China) following the manufacturer’s instructions and expressed as mmol per gram of frozen weight (FW).

### 2.5. Determination of Lipid Peroxidation 

Lipid peroxidation was determined by monitoring the malondialdehyde (MDA) content in both CEO treated or control peel samples following the method of Chen et al. [27], and expressed as mmol·g^‒1^ FW (frozen weight).

### 2.6. Assay of Defense-related Enzymes Activities

Frozen peel samples (2.0 g fresh weight, stemmed from the CEO treated or control fruits) were homogenized with 5 mL of different ice-cold extraction buffers to assay of the following defense-related enzymes. 100 mM ice-cold sodium acetate buffer (1 mM EDTA, ethylenediamine tetraacetic acid, 5 mM β-mercaptoethanol, 1% AsA, pH 5.2) for β-1,3-glucanase (β-Glu, EC 3.2.1.73) and chitinase (CHI, EC 3.2.1.14) assays; 50 mM ice-cold Tris-HCl buffer (15 mM β-mercaptoethanol, 5 mM EDTA, 5 mM AsA, 1 mM PMSF, phenylmethylsulfonyl fluoride, 0.15% PVP, polyvinylpyrrolidone, pH 8.8) for phenylalanine ammonia-lyase (PAL, EC 4.3.1.5) assay; 100 mM ice-cold sodium acetate buffer (1 mM PEG, polyethylene glycol 4% PVP, 1% Triton X-100, pH 5.5) for peroxidase (POD, EC 1.11.1.7) and polyphenol oxidase (PPO, EC 1.10.3.1) assays; 100 mM ice-cold phosphate buffer (4% PVP, 1% Triton X-100, pH 6.8) for lipoxygenase (LOX, EC 1.13.11.12) assay. All homogenates were centrifuged at 12,000 × g for 30 min (Hettich Universal-320R), and then supernatants obtained were used for assay of defense-related enzymes activities. 

The β-Glu activity was determined referring to the method of Abeles et al. [28] with slight modifications. 100 μL of enzyme solution was incubated in a tube with 100 μL of 0.4% (*w/v*) laminarin (Sigma-Aldrich, St.Louis, MO, USA) at 37 °C for 40 min and then quickly add 1.8 mL distilled water and 1.5 mL 3, 5-dinitrosalicilate to each tube. Subsequently, the solution was boiled in water for 5 min to terminate the reaction, after which it was diluted to 25 mL with distilled water. The absorbance was measured at 540 nm (T6, Beijing Purkinje General Inst. Co. Ltd., Beijing, China) using glucose (1 mg/mL) as the standard solution. One unit of β-Glu activity was defined as the amount of enzyme that produced 1 μM of glucose per hour and expressed as U·h^−1^·g^−1^ FW.

Chitinase (CHI) activity was assayed using chitinase (Sigma-Aldrich) as the substrate. To perform CHI activity assay, the reaction mixture consisted of 0.5 mL of enzyme solution, 0.5 mL of 50 mM sodium acetate buffer (pH 5.2) and 0.1% (*w/v*) chitinase was incubated at 37 °C for 1 h. Then, 100 μL of 0.3 (*w/v*) desalting snailase was added to the above reaction solution and further incubated at 37 °C for 1 h. The enzyme reaction was terminated by adding 200 μL of 0.6 M potassium tetraborate and boiling in water for 3 min. The absorbance was measured at 585 nm (T6, Beijing Purkinje General Inst. Co. Ltd.) using N-acetylglucosamine (0.1 mM) as the standard solution. One unit of CHI activity was defined as the amount of enzyme that produced 1 μM of N-acetylglucosamine per hour and expressed as U·h^−1^·g^−1^ FW.

Phenylalanine ammonia-lyase (PAL) activity was detected by a commercial PAL assay kit (Jiancheng Bioeng. Inst.) in accordance with manufacturer’s instructions. One unit of PAL activity was defined as an increment in absorbance of 0.01 at 290 nm (Shimadzu UV-2600, kyoto, Japan) per hour, and expressed as U·h^−1^·g^−1^ FW. 

Peroxidase (POD) activity was determined following the method of guaiacol oxidation at 470 nm by monitoring the increase in absorbance at 470 nm (Shimadzu UV-2600) in the reaction mixture consisting of 200 μL of enzyme solution and 3.0 mL of 25 mM guaiacol in sodium acetate buffer (50 mM, pH 5.5), which was activated by addition of 200 μL of 0.5 M H_2_O_2_ in sodium acetate buffer (50 mM, pH 5.5). One unit of POD activity was defined as an increment of 0.01 in absorbance at 470 nm due to guaiacol oxidation in the presence of H_2_O_2_, and expressed as U·min^−1^·g^−1^ FW.

Polyphenol oxidase (PPO) activity was determined based on the decomposition of catechol. To achieve this, 0.5 mL of enzyme solution and 1.0 mL of 50 mM catechol in sodium acetate buffer (100 mM, pH 5.5) was added to 4.0 mL of sodium acetate buffer (50 mM, pH 5.5). After 15 s, the absorbance was recorded at 420 nm every 1 min for 5 min. One unit of PPO activity was defined as an increment of 0.01 in absorbance at 420 nm per hour due to decomposition of catechol, and expressed as U·h^−1^·g^−1^ FW.

Lipoxygenase (LOX) activity was determined following the method of Surrey Kenneth [29]. Firstly, the substrate was mixed with 2.7 mL of sodium phosphate buffer (100 mM, pH 6.8) and 100 μL of 0.5% (*v/v*) sodium soyate (Sigma-Aldrich), and incubated at 30 °C for 15 min. The enzyme reaction was activated by adding 200 μL of enzyme solution, and the absorbance at 234 nm (Shimadzu UV-2600) was recorded every 15 sec for 3 min. One unit of LOX activity was defined as an increment in absorbance of 0.01 at 234 nm, and expressed as U·min^−1^·g^−1^ FW.

### 2.7. Gene Expression Analysis by Real Time-Quantitative Polymerase Chain Reaction

#### 2.7.1. Total RNA Extraction and cDNA Synthesis

High quality total RNA was independently extracted from peel samples stemmed from the CEO treated or control citrus fruits according to the method described by Ballester et al. [30] and dissolved in RNase-free water. The total RNA extracted were quantified using a Micro Spectrophotometer (NanoDrop 1000, Thermo-Fisher, Waltham, MA, USA) and confirmed by agarose gel electrophoresis. Following the manufacturer’s instructions, a PrimeScript RT reagent kit (TaKaRa, Kyoto, Japan) was applied for synthesizing the first-strand cDNA used as the template to perform the real time-quantitative PCR analysis.

#### 2.7.2. Primer Design

Based on the target genes (β-Glu, CHI, PAL, POD, PPO, LOX, β-tubulin) sequences from the GENBANK database, the primers for the chosen genes were designed with the primer premier 6.0 software (Premier Biosoft International, Palo Alto, CA, USA) and listed in Table 1. Citrus β-tubulin was used as the reference gene for normalizing the expression of each sample.

#### 2.7.3. Real Time-Quantitative Polymerase Chain Reaction Analysis

The RT-qPCR was performed in 96-well PCR plates using BIO-RAD CFX96 Real-Time PCR Detection System (Bio-Rad, Hercules, CA, USA) under the following procedures: pre-denaturation at 95 °C for 30 s, followed by 40 cycles at 95 °C for 5 s, 60 °C for 10 s and 72 °C for 15 s. Polymerase chain reaction amplification was carried out in a total volume of 25 μL, which contained 12.5 μL of SYBR (TaKaRa), 1.0 μL of each primer (10 μmol/L), 2.0 μL of cDNA and 9.5 μL of RNase-free water. The melting curve analysis was performed at over the end of each PCR reaction from 60 °C to 95 °C. The relative RNA expression was calculated using the 2^−ΔΔCt^ method [31]. 

### 2.8. Statistical Analysis

The experimental data were calculated and expressed as the means of three replicated samples ± standard errors (S.E.). The differences between CEO-treated and control group were analyzed by Tukey honest significant difference (HSD) test using the SPSS version 17.0 (SPSS Inc., Chicago, IL, USA) to estimate if differences were considered significant at levels of *p* < 0.05.

## 3. Results and Discussion

### 3.1. Effect of Clove Essential Oil for Inhibiting Disease Development of Blue Mold Caused by Penicillium italicum

A disease development of blue mold infection in CEO-treated fruits at different concentrations (0, 0.05, 0.1, 0.2, 0.4 and 0.8%, *v/v*) during post inoculation is shown in Table 2. It was remarkable to note that treatment with different concentration gradients of CEO significantly (*p* < 0.05) reduced the lesion diameter (F values were 144.00, 391.13 and 783.55 at 3, 5 and 7 dpi, respectively) and disease severity (F values were 80.49, 71.04 and 70.09 at 3, 5 and 7 dpi, respectively) in citrus fruit inoculated with *P. italicum.* At the end of inoculation (7 dpi), the lesion diameters were 56.1 mm, 47.8 mm, 38.4 mm, 29.2 mm and 26.8 mm, and the disease severity rates were 98.8%, 95.0%, 80.0%, 66.3% and 63.1% compared with those of control fruit after CEO treatment at 0.05, 0.1, 0.2, 0.4 and 0.8%, respectively (Table 2). Furthermore, the disease severity had no significant difference (F values were 1.62, 3.39 and 1.00) by applying 0.4% and 0.8% CEO treatment at 3, 5 and 7 dpi, but had significant difference with 0.05%, 0.1% and 0.2% CEO treatment (*p* < 0.05).

The presence of tender peels and nutritious flesh in *Citrus reticulate* fruit make it highly susceptible to various fungal diseases caused by *P. italicum* [32]. Recently, one of our studies showed that CEO has significant in vitro antifungal activity against *P. italicum* in citrus fruits, and the growth inhibitory effect was positively correlated with CEO in a concentration-dependent manner [9]. In vivo experiments herein, both lesion diameter and disease severity of citrus fruit infected with *P. italicum* were significantly reduced with an increasing CEO concentration (Table 2). This indicated that CEO also displayed antifungal activity under in vivo conditions. Additionally, the results showed that CEO treatment at the concentration of 0.4% (*v/v*) effectively lessened the lesion diameter and observably reduced the disease severity of blue mold rot. Limited data has reported the antifungal activity of CEO in citrus fruit and other horticultural products, while pioneering results of the current study showed a prominent control efficacy of blue mold caused by *P. italicum* in *C. reticulate*. This control efficacy of CEO in controlling postharvest blue mold was also observed by Regnier et al. [33] and Jhalegar et al. [34] applying CEO treatment to protect ‘Valencia’ orange and ‘Kinnow’ mandarin from *P. digitatum* infection. These results were highly in agreement with the previous reports that the use of plant essential oils could reduce postharvest various diseases of citrus and other fruits [17,19,20,23,34,35]. Therefore, the application of CEO would provide a promising approach to help control blue mold disease development and reduce postharvest fungal rots loss of harvested citrus fruit.

### 3.2. Effect of Clove Essential Oil Treatment on H_2_O_2_ Content in Citrus Fruit

**Figure 1:**
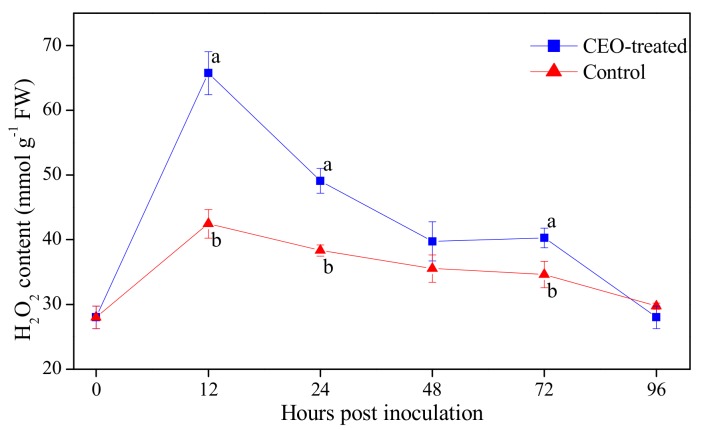
H_2_O_2_ content in citrus fruit treated with CEO (0.4%, *v/v*) infected with *P. italicum*, and incubated at 25 °C for 96 h. Each value represents the mean of three replicates ± standard error (S.E.). Different lowercase letters indicate significant differences (*p* < 0.05) between CEO-treated and control group at the same time by Tukey HSD test.

H_2_O_2_ content in both CEO-treated and control fruits exhibited a peak level initially at 12 hpi and then declined towards the end of incubation (Figure 1). The CEO treatment significantly (*p* = 0.001, 0.005 and 0.018 at 12, 24 and 72 hpi, < 0.05) disrupted the rise and delayed the decline in H_2_O_2_ levels, which remained significantly higher (*p* < 0.05) in CEO-treated fruits than in control fruits during the entire incubation period.

Plant motivated H_2_O_2_ as a signaling molecule for inducing host-defense responses in fruit hosts was infected by postharvest pathogenic fungus [36]. Besides its direct in vitro antifungal activity, CEO also has the potential ability to reduce the incidence rate of fungus rots [9,26]. This study showed that citrus fruit wounds treated with CEO at 0.4% significantly presented the accumulation in H_2_O_2_ production (Figure 1). The higher H_2_O_2_ accumulation that contributed to CEO treatment might be an important phenomenon in the defense response and could play a role in halting the incidence of blue mold disease in citrus fruit. In line with our data, Abouraïcha et al. [37] demonstrated that a rapid accumulation of H_2_O_2_ in oligoulvans-treated apple was tightly linked to postharvest disease resistance. Thus, we found that a high stimulation in H_2_O_2_ production as the result of CEO treatment stimulation was required for inducing disease resistance in citrus fruit.

### 3.3. Effect of Clove Essential Oil Treatment on Malondialdehyde Content in Citrus Fruit

**Figure 2:**
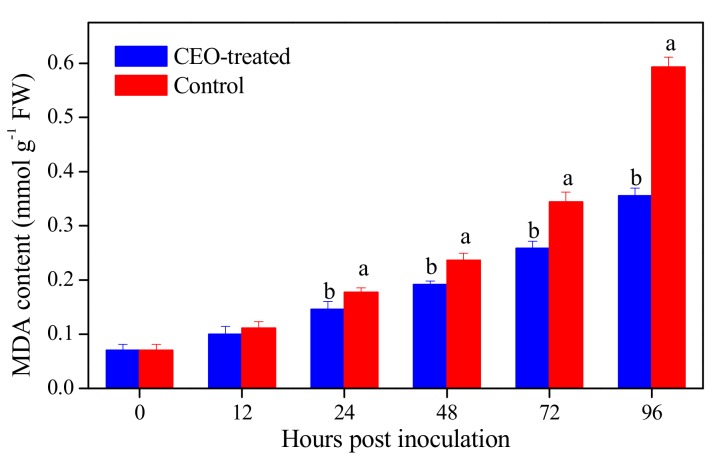
Malondialdehyde (MDA) content in citrus fruit treated with CEO (0.4%, *v/v*) infected with *P. italicum*, and incubated at 25 °C for 96 h. Each value represents the mean of three replicates ± standard error (S.E.). Different lowercase letters indicate significant differences (*p* < 0.05) between CEO-treated and control group at the same time by Tukey HSD test.

**Figure 3:**
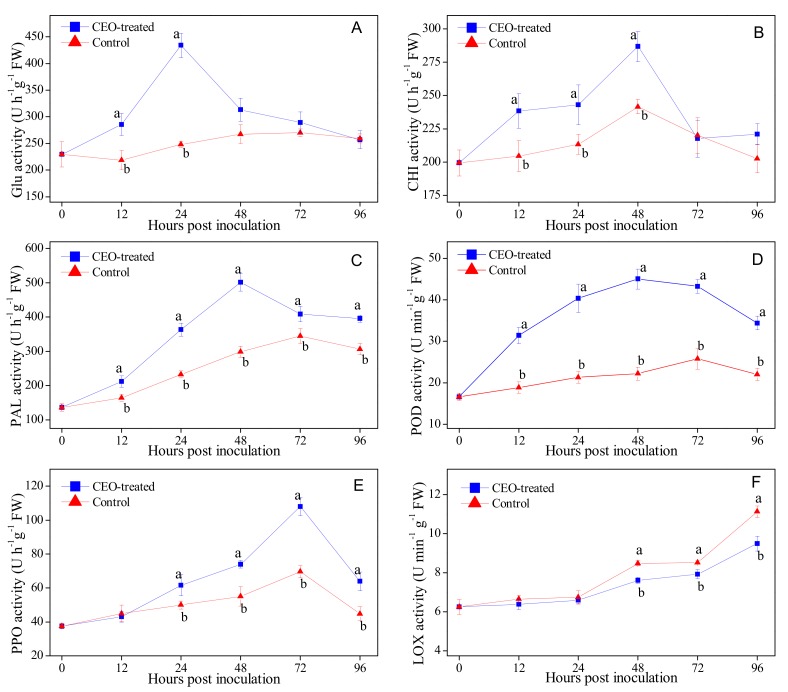
The activities of β-Glu (**A**), CHI (**B**), PAL (**C**), POD (D), PPO(**E**) and LOX (**F**) in citrus fruit treated with CEO (0.4%, *v/v*) infected with *P. italicum*, and incubated at 25 °C for 96 h. Each value represents the mean of three replicates ± standard error (S.E.). Different lowercase letters indicate significant differences (*p* < 0.05) between CEO-treated and control group at the same time by Tukey HSD test.

As shown in Figure 2, the malondialdehyde (MDA) content in both CEO-treated and control fruit gradually increased during the incubation. The CEO treatment significantly (*p* = 0.030, 0.006, 0.003 and 0.001 at 24, 48, 72 and 96 hpi, < 0.05) delayed the accumulation of MDA content in citrus fruit. The MDA content in CEO-treated fruit was 0.356 mmol g^−1^, which was 40.01% lower than that in the control group at the end of incubation. Malondialdehyde (MDA) is used as a marker to evaluate the level of lipid peroxidation and cell membrane damage [27]. Clove essential oil might reduce MDA content (Figure 2) through increasing defense-related enzymes activities, reducing LOX activity (Figure 3) and enhancing resistance against blue mold decay caused by *P. italicum* in citrus fruit, as has been reported in raspberry [38] and sweet pepper [39] treated with lemon verbena and cinnamon essential oil, respectively. Lower MDA content in CEO-treated fruit could be attributed to the inductive effect of CEO treatment through activating defense-related enzymes. Therefore, based on our result and previous research, the application of EOs could decease fungal decay in citrus fruit or other horticultural crops through controlling the membrane lipid peroxidation.

### 3.4. Effects of Clove Essential Oil Treatment on the Activities of Defense-related Enzymes in Citrus Fruit

The CEO treatment induced the activities of defense-related enzymes in citrus fruit inoculated with *P. italicum* of incubation at 25 °C (Figure 3). As shown in Figure 3A, the β-Glu activity in CEO-treated fruit was significantly higher (*p* = 0.047 and 0.004 at 12 and 24 hpi, < 0.05) than the control group in the initial and interim time of incubation, and reached a peak value of 434.18 U·h^−1^·g^−1^ at 24 hpi, which shown 1.75 times as the control group (248.27 U·h^−1^·g^−1^) at the same time point. The CHI activity increased in both the CEO-treated and control fruit and reached its highest value at 48 hpi, but the CHI activity in the CEO-treated fruit was higher than the control fruit before 72 h of incubation (Figure 3B). The activities of PAL and POD in the CEO-treated and control fruit increased and reached their peaks at 72 hpi and 48 hpi, respectively, followed by a gradual decrease. CEO treatment significantly (*p* < 0.05) promoted the increase in the activities of PAL and POD during the entire incubation period (Figure 3C,D). The PPO activity in the CEO-treated fruit was significantly (*p* = 0.036, 0.010, 0.001 and 0.028 at 24, 48, 72 and 96 hpi, < 0.05) higher than the control group throughout the entire incubation period except the first 12 hpi and peaked at 72 hpi, which was 1.55 times higher than that in the control group (Figure 3E). The LOX activity in both the CEO-treated and control fruit increased with incubation time, but the lower LOX activity in the CEO-treated fruit was determined starting at 48 hpi and stayed at a low level during the remainder of the incubation process (Figure 3F).

Pathogenesis related (PR) proteins are a large group of proteins that have important functions in plant defense responses to pathogen attacks and other physiological stresses (including chilling, salinity, drought, heavy metals, etc.) [40]. Both β-Glu and CHI are the main PR members and are known to be considered the important marker enzymes that may directly inhibit pathogens growth by decomposing β-1,3-glucan and chitin, which are two of the major constituents of fungal cell wall [19]. Generally, plant disease resistance is accompanied by prominent activation in the two above enzyme activities. Among PR proteins, PAL, POD and PPO were reported to be associated with disease resistance in plants and participated in the biosynthesis and oxidation of phenolic compounds [30,41]. Phenylalanine ammonia-lyase (PAL) and POD are the two key enzymes in the phenylpropanoid metabolic pathway to induce the synthesis, while PPO plays an important role in active oxygen metabolism in plant and oxides phenolic compounds into toxic quinones for inhibiting pathogens growth. Lipoxygenase (LOX) is the most critical enzyme that has an important influence on the synthesis of volatile esters in plant tissue [42]. According to the results obtained, the activities of these defense-related enzymes (β-Glu, CHI, PAL, POD, PPO and LOX) were more profoundly enhanced or reinforced in the CEO-treated fruit after *P. italicum* infection (Figure 3), along with a lower disease severity of blue mold caused by *P. italicum* in CEO-treated citrus fruits (Table 1). It worth noting that the CEO treatment induced the increase of defense-related enzymes activities that coincided with lower MDA content in citrus fruit (Figure 2 and Figure 3). Many induced factors, as plant essential oil, were effective in controlling various postharvest fungal diseases by activating PR proteins. Higher phenylpropanoid metabolic pathway activities representing higher enzyme activity of PAL, POD, and PPO in CEO-treated fruit give rise to higher phenolic compounds accumulation. This in turn leads to a lower MDA content and LOX activity, that may enhance the activity of fruit defense mechanisms by priming defense responses. For example, thyme essential oil fumigation enhanced the activities of β-Glu and CHI in avocado fruit for the controlling of anthracnose caused by *Colletotrichum gloeosporioides* Penz. [19]. Tea tree oil vapor treatment significantly enhanced the disease resistance of postharvest strawberry fruit against *Botrytis cinerea* through the induction of five defense-related enzymes, such as PAL, POD, PPO, β-Glu and CHI [24]. Likewise, *Aloe vera* gel treatment induced the activities of defense-related enzymes, including LOX, PAL and POD in ’Shogun’ mandarins inoculated with *P. digitatum*, the causal agent of postharvest green mold in citrus fruit [43]. Thus, CEO treatment may induce citrus fruit resistance against *P. italicum* through a pathogen-dependent priming mechanism.

### 3.5. Effects of Clove Essential Oil Treatment on Defense-related Gene Expression in Citrus Fruit

**Figure 4:**
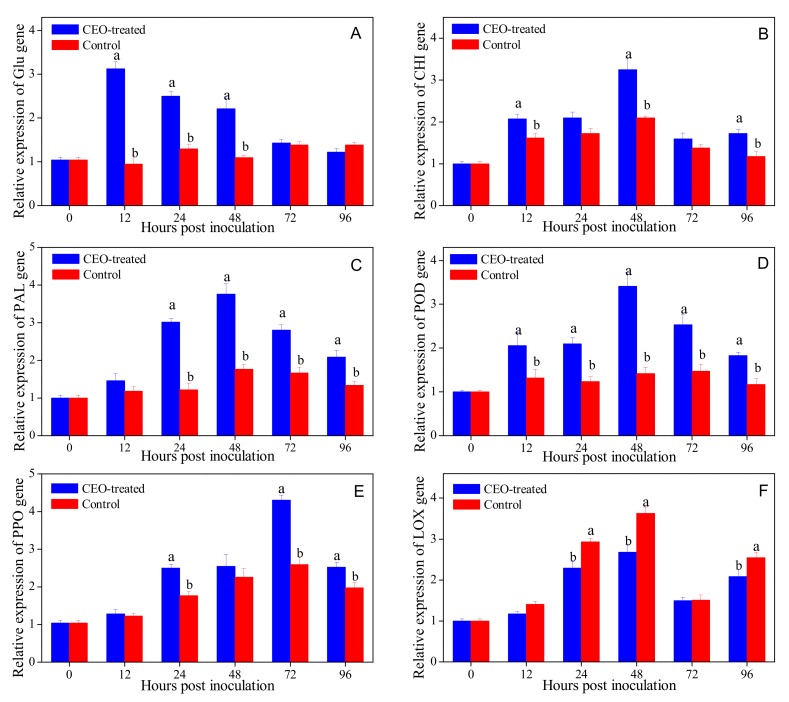
Relative expression of β-Glu (**A**), CHI (**B**), PAL (**C**), POD (**D**), PPO (**E**) and LOX (**F**) genes in citrus fruit treated with CEO (0.4%, *v/v*) infected with *P. italicum*, and incubated at 25 °C for 96 h. Each value represents the mean of three replicates ± standard error (S.E.). Different lowercase letters indicate significant differences (*p* < 0.05) between CEO-treated and control group at the same time by Tukey HSD test.

The transcript levels of six defense-related genes encoding β-Glu, CHI, PPO, POD, PAL and LOX were analyzed by using RT-qPCR and shown in Figure 4. The pattern of expression was studied in both CEO-treated and control flavedo tissues at 12, 24, 36, 48, 72 and 96 hpi. The expression level of β-Glu gene in CEO-treated fruit was up-regulated 3.30-fold compared with the control at 12 h (Figure 4A). A similar pattern of expression levels of CHI, PAL and POD genes in CEO-treated fruit were up-regulated by 3.25, 2.76 and 3.42-fold, when compared with their levels in control fruits at 48 hpi, respectively (Figure 4B–D). In addition, CEO treatment induced the up-regulated expression of PPO gene gradually increasing from 12 hpi and reaching the maximum expression level at 72 hpi (Figure 4E). However, the gene expression of LOX showed a 35.6% decrease at 48 hpi in CEO-treated fruit (Figure 4F).

To further illuminate the possible defense mechanism involved, this study investigated the transcriptomics responses of CEO treatment on induced PR genes of citrus fruit infected with *P. italicum*. In the present study, there is an interesting phenomenon where the expression level of β-Glu occurred at 12 hpi, which was earlier than the up-regulations of inducible resistance genes, including CHI, PAL, POD, PPO and LOX. In addition, the activities and expression levels of these defense-related enzymes were more enhanced or reinforced in the CEO-treated fruit after *P. italicum* infection, when compared with the control group (Figure 3 and Figure 4). The transcription levels of PAL, POD, and PPO directly associated with the phenylpropanoid metabolic pathway in CEO-treated fruit were lower than that of the control group. In present results, up-regulated expression of defense-related enzymes may contribute to enhance the fruit disease-resistance and reduce the incidence of postharvest fungal disease of citrus fruit. A recent report has shown a similar result where thyme essential oil reduced anthracnose disease incidence via up-regulated expression of the PAL gene and down-regulated expression of the LOX gene in ‘Hass’ and ‘Ryan’ avocado fruit [42]. The defense mechanism of postharvest CEO treatment exhibited in the present work is in agreement with the previous reports regarding the gene expressions of defense-related enzymes in cyclic lipopeptides-treated ’Valencia’ orange fruit [44] and *Aloe vera* gel-treated ’Shogun’ mandarins [43], as well as in thyme and savory essential oil treated apples [22] subjected to fungal infections. 

## 4. Conclusions

The current study suggested that CEO treatment showed a significant impact on the growth of blue mold in citrus fruits caused by *P. italicum* during incubation at 25°C. The possible mechanism of CEO treatment in controlling blue mold in postharvest citrus fruit was thought to be linked with the rise in H_2_O_2_ levels, the suppression of MDA accumulation, and the induction of built-in cellular defense-associated enzymes. Taken together, the results of the current study will provide an alternative strategy using CEO for citrus fruits protection against postharvest fungal decay problems.

## Figures and Tables

**Table 1:** Primers used for real time-quantitative polymerase chain reaction (RT-qPCR) of key defense-related genes in citrus fruit.

Target Gene	GenBank Accession	Forward Primer (5′–3′)	Reverse Primer (5′–3′)
β-Glu	AY971953	ACCTCCGAAGAATCGCTTCCAA	TGTTTCTCATGGCGGGAACA
CHI	AF090336	AATGATGAACGATGCCCTGCCA	CCACTTGATGCTGTCTCCAA
PAL	DQ088064.1	GATTACGGATTCAAGGGTGC	TTGGTGACAGGATTGGCGAG
POD	AJ582678.1	AGCCAGGAGACAATGAACAG	TAGTTTCATGGCCAGTTTGGGC
PPO	XM_006468155	AACTGCTGCCCACCAAAATC	GGTCATAAGCCCCATCACAATA
LOX	XM_006483993	TGACCAAGTCCAAGTTCTGCC	AACGTCTTTTGCGTCCACC
β-tubulin	AF052608	GGTGCAAATCCCACCATGAA	TGGTGTCACTTGCTGCTGCCTGA

β-Glu: β-1,3-glucanase, CHI: chitinase, PAL: phenylalanine ammonia-lyase, POD: peroxidase, PPO: polyphenol oxidase, and LOX: lipoxygenase.

**Table 2:** Lesion diameter and disease severity evolution in citrus fruit treated with different concentrations of clove essential oil (CEO) infected with *P. italicum* at different days post inoculation (dpi).

Index	DPI	Concentrations of CEO (%, *v/v*)					
		0.0	0.05	0.1	0.2	0.4	0.8
**Lesion diameter**	3	23.3 ± 1.33 a	19.9 ± 0.99 b	16.4 ± 1.07 c	13.8 ± 0.92 d	11.7 ± 1.57 e	9.8 ± 2.10 f
	5	35.6 ± 1.71 a	32.2 ± 1.03 b	27.6 ± 1.26 d	22.7 ± 1.16 d	17.6 ± 1.07 e	13.9 ± 1.66 f
	7	58.9 ± 1.66 a	56.1 ± 1.20 b	47.8 ± 1.48 c	38.4 ± 1.71d	29.2 ± 1.40 e	26.8 ± 1.69 e
**Disease severity**	3	46.9 ± 4.27 a	42.5 ± 5.40 ab	33.8 ± 3.23 b	21.3 ± 2.88 c	8.8 ± 3.23 d	5.0 ± 3.53 d
	5	69.4 ± 5.15 a	62.5 ± 5.40 a	58.8 ± 6.61 ab	48.1 ± 4.27 b	23.8 ± 4.27 c	22.5 ± 2.88 c
	7	100 ± 0.00 a	98.8 ± 2.50 a	95.0 ± 4.56 a	80.0 ± 4.08 b	66.3 ± 3.23 c	63.1 ± 6.25 c

Each value is the means of four replicates ± standard error (S.E.). Means within row with the different letters (a, b, c, d, e, and f, respectively) for each time point (day 3, day 5 and day 7) indicate significant difference (*p* < 0.05) among different concentrations of CEO by Tukey HSD test.
